# A template and tutorial for preregistering studies using passive smartphone measures

**DOI:** 10.3758/s13428-024-02474-5

**Published:** 2024-08-07

**Authors:** Anna M. Langener, Björn S. Siepe, Mahmoud Elsherif, Koen Niemeijer, Pia K. Andresen, Samir Akre, Laura F. Bringmann, Zachary D. Cohen, Nathaniel R. Choukas, Konstantin Drexl, Luisa Fassi, James Green, Tabea Hoffmann, Raj R. Jagesar, Martien J. H. Kas, Sebastian Kurten, Ramona Schoedel, Gert Stulp, Georgia Turner, Nicholas C. Jacobson

**Affiliations:** 1 Groningen Institute for Evolutionary Life Sciences, University of Groningen, Groningen, The Netherlands; 2 Department of Psychometrics and Statistics, Faculty of Behavioural and Social Sciences, University of Groningen, Groningen, The Netherlands; 3 Psychological Methods Lab, Department of Psychology, University of Marburg, Marburg, Germany; 4 Department of Psychology, University of Birmingham, Birmingham, UK; 5 Faculty of Psychology and Educational Sciences, KU Leuven, Louvain, Belgium; 6 Department for Methodology and Statistics, Utrecht University, Utrecht, The Netherlands; 7 Medical Informatics Home Area, University of California, Los Angeles, CA USA; 8 Interdisciplinary Center Psychopathology and Emotion Regulation, (ICPE), University Medical Center Groningen, University of Groningen, Groningen, The Netherlands; 9 Department of Psychology, University of Arizona, Tucson, AZ USA; 10 Division of Child and Adolescent Psychiatry, Department of Psychiatry, Lausanne University Hospital, Lausanne, Switzerland; 11 School of Clinical Medicine, University of Cambridge, Cambridge, UK; 12 School of Allied Health, Physical Activity for Health Research Centre, Health Research Institute, University of Limerick, Limerick, Ireland; 13 Department of Marketing, Faculty of Economics and Business, University of Groningen, Groningen, The Netherlands; 14 Department of Planning, Faculty of Spatial Sciences, University of Groningen, Groningen, The Netherlands; 15 Department of Interdisciplinary Social Science, Utrecht University, Utrecht, The Netherlands; 16 Charlotte Fresenius Hochschule, University of Psychology, Munich, Germany; 17 Department of Psychology, Ludwig-Maximilians-Universität München, Munich, Germany; 18 Department of Sociology & Inter-University Center for Social Science Theory and Methodology, Grote Rozenstraat 31, 9712 TS Groningen, The Netherlands; 19 Center for Technology and Behavioral Health, Geisel School of Medicine, Dartmouth College, Lebanon, NH USA; 20 Department of Psychiatry, Geisel School of Medicine, Dartmouth College, Lebanon, NH USA; 21 Department of Computer Science, Dartmouth College, Lebanon, NH USA; 22 Faculty of Science and Engineering, Nijenborgh 7, 9747 AG Groningen, The Netherlands

**Keywords:** Preregistration, Digital phenotyping, Open science, Reproducibility, Ambulatory assessment, Transparency, Mobile sensing

## Abstract

Passive smartphone measures hold significant potential and are increasingly employed in psychological and biomedical research to capture an individual's behavior. These measures involve the near-continuous and unobtrusive collection of data from smartphones without requiring active input from participants. For example, GPS sensors are used to determine the (social) context of a person, and accelerometers to measure movement. However, utilizing passive smartphone measures presents methodological challenges during data collection and analysis. Researchers must make multiple decisions when working with such measures, which can result in different conclusions. Unfortunately, the transparency of these decision-making processes is often lacking. The implementation of open science practices is only beginning to emerge in digital phenotyping studies and varies widely across studies. Well-intentioned researchers may fail to report on some decisions due to the variety of choices that must be made. To address this issue and enhance reproducibility in digital phenotyping studies, we propose the adoption of preregistration as a way forward. Although there have been some attempts to preregister digital phenotyping studies, a template for registering such studies is currently missing. This could be problematic due to the high level of complexity that requires a well-structured template. Therefore, our objective was to develop a preregistration template that is easy to use and understandable for researchers. Additionally, we explain this template and provide resources to assist researchers in making informed decisions regarding data collection, cleaning, and analysis. Overall, we aim to make researchers' choices explicit, enhance transparency, and elevate the standards for studies utilizing passive smartphone measures.

In recent years, there has been a growing trend in utilizing digital phenotyping to capture and analyze individuals' behavior. Digital phenotyping involves the real-time and moment-by-moment quantification of an individual's behavioral patterns using data collected from personal digital devices (Torous et al., 2016). In particular, smartphones are capable of continuously capturing a wide range of data sources, including GPS locations, Wi-Fi connections, calls, text messages, movement patterns, and app usage throughout a study. The use of passive measures is promising due to their ability to both minimize participant burden and capture behavior in a real-world context. This makes them particularly valuable for researchers seeking to understand behavioral patterns in natural settings, and thus can provide insights that complement laboratory-based experiments. Overall, passive measures are expected to transform health care, education, and clinical practice (Huckvale et al., 2019; Jongs, 2021; Seiferth et al., 2023; Velozo et al., 2022), providing new opportunities to study phenomena relevant to a wide range of research fields.

The promise of implementing digital phenotyping methods at a large scale across different contexts and fields of study comes with a growing need for guidance on what counts as best practice. Passive measures present numerous methodological challenges, particularly in the areas of data collection, cleaning, and analysis (Davidson, 2022; Hicks et al., 2019; Huckvale et al., 2019; Onnela, 2021; Velozo et al., 2022). Even before data collection begins, researchers must conceptualize the constructs they are interested in studying, which can be particularly challenging in digital phenotyping studies where constructs are often poorly defined (Huckvale et al., 2019; Langener et al., 2023; Mohr et al., 2017). Additionally, researchers face critical decisions when selecting the device and app to capture individuals' behavior (Davidson, 2022; Velozo et al., 2022). Further, due to the large amount of data generated, thorough data-cleaning procedures are necessary (Hicks et al., 2019). Lastly, often more computationally intensive methods, such as machine learning models, are used, which require various analytical decisions. For example, researchers must determine how to divide data into training and test sets and how to tune hyperparameters to optimize model performance (Yang & Shami, 2020).

Previous research has shown that different decisions made during the research process can result in different conclusions. For instance, the choice of a specific time scale for data summarization and analysis has been shown to influence the accuracy of prediction (Bos et al., 2019; Cai et al., 2018; Heijmans et al., 2019; Langener et al., 2024a, 2024b; Sun et al., 2023). Moreover, the method chosen for data preprocessing and different modeling decisions can impact, for example, the accuracy of predicting sleep quality, mood, and depression (Niemeijer et al., 2022). Hence, it is essential to transparently document the choices made in order to ensure the reproducibility of findings (Wrzus & Schoedel, 2023).

Implementing open science practices in digital phenotyping can improve transparency, reproducibility, and replication. Specifically, preregistering study design and analysis plan encourages researchers to make informed decisions and report their analysis choices and findings (Nosek et al., 2018). Preregistration involves registering research questions, analysis plans, and analytical steps in advance, ensuring that these choices are not influenced by the study outcomes (Nosek et al., 2018). This process aids in identifying analyses that were planned a priori versus those conducted post hoc (Nosek et al., 2019). Moreover, preregistration offers the advantage of detecting questionable research practices and minimizing the impact of publication bias by making all studies discoverable (Nosek et al., 2019). In addition, the effort involved in planning a study and preregistering those plans may reduce the likelihood of problems arising later while conducting the study and analysis. Unfortunately, despite the numerous benefits, preregistration is rarely practiced in studies utilizing passive smartphone measures. However, recently, researchers have started to point out that preregistration is an important way to enhance the usefulness of digital phenotyping studies (Davidson, 2022; Velozo et al., 2022).

The limited adoption of preregistration might be attributed, in part, to the challenge of anticipating all of the forking paths in the vast garden of digital phenotyping research. Unlike other research fields, where templates exist to assist researchers in considering specific decisions ahead of time (e.g., Kirtley et al., 2021), such a template is currently missing for digital phenotyping studies. This absence creates a hurdle for researchers who want to preregister their study plans.

In this paper, we present an accessible and user-friendly preregistration template that guides researchers in making informed decisions regarding data collection, cleaning, and analysis. The purpose of this tutorial paper is to serve as a narrative companion guide, explaining the considerations and providing further information behind the main topics for each section of the preregistration template. For each topic, we aim to emphasize the importance of transparent communication of how a given smartphone sensing study will be conducted and the reasoning behind it. We specifically focus on research that uses passive smartphone measures. However, we believe that this template is easily adaptable for other passive data types, for example, coming from wearable devices such as smartwatches, pollution sensors, specific devices for measuring physiological parameters (heart rate, insulin), or research-grade accelerometers. It may also be a useful guide for passive non-worn data collection devices, from vehicle/door counters to GPS devices mounted on bicycles to digital medicine bottle caps. By lowering the barriers for preregistration, we aim to promote normative change toward sound scientific conduct among our research community who will embrace smartphone sensing methods over the next decades.

## Process of developing a preregistration template

The development of the preregistration template began during a Hackathon named “Developing a template and tutorial for preregistering studies using passive smartphone measures” organized at the Society for the Improvement of Psychological Science Conference 2023. A review of the existing literature was conducted, focusing on the challenges encountered when working with passive smartphone measures (e.g., Davidson, 2022; Hicks et al., 2019; Onnela, 2021; Velozo et al., 2022). Additionally, we incorporated literature that provides guidelines for the use of machine learning models, which are commonly employed in studies utilizing digital phenotyping (Collins et al., 2015). We were inspired by existing preregistration templates, such as the template designed for preregistering experience sampling method (ESM) studies (Kirtley et al., 2021), and aimed to extend them for digital phenotyping studies.

## Proposed core elements of a preregistration template for studies using passive smartphone measures

In the remaining sections of this paper, we elaborate on the decisions that need to be made when dealing with passive smartphone measures, which are also incorporated in our preregistration template. More specifically, we review each section and corresponding decisions required when filling out the template, providing explanations of their importance and the considerations that need to be addressed when making them. Our focus is on the sections and elements that hold particular importance for studies utilizing passive smartphone measures; thus, some elements are only briefly mentioned. A comprehensive version of the template and examples can be found on our OSF page: https://osf.io/jy7xg/. Researchers who want to use this template are invited to complete each section that applies to their research and then upload the template as an “open-ended” registration in OSF.^1^ We discuss six core themes that are important when working with passive smartphone measures, which also form the basis of our template's structure: the (1) conceptualization of constructs and research question, (2) data collection and sampling plan, (3) data processing and feature engineering, (4) missing data handling, (5) data analyses, and (6) replicability and open science practices.

### Conceptualization and research question

Before a study begins, researchers must make decisions about the design of their study. This section focuses on formulating a clear research question and deciding on different research designs (e.g., exploratory vs. confirmatory, idiographic vs. nomothetic). We also consider the specification and operationalization of key constructs (see Textbox 1 for all proposed elements in our template, with those discussed in greater detail in this paper highlighted in bold).

**Textbox 1** Proposed elements to preregister in the section “Conceptualization and research question”.

**Table:** 

• Study information and research question
o Title
o Authors
o Subject/discipline
o **Study purpose and (well-defined) research question**
o Hypotheses
o Study type and blinding
o **Study design**
o Rationale for using passive sensing
• Operationalization of main constructs
o **Main constructs of interest**
o **Assumptions**

Bold topics are further detailed in this tutorial paper.

#### Study information and research question

##### Study purpose and (well-defined) research question

Providing clear information about the study aim and formulating a well-defined research question is useful in all studies, but particularly in digital phenotyping studies, which often gather large amounts of data. The research aim can fall into two primary categories: confirmatory, which involves the testing of specific hypotheses often using inferential statistics, or exploratory, where the objective is to find new patterns in the data, subsequently leading to the generation or modification of hypotheses, models, and theories (Höfler et al., 2022). In exploratory approaches, researchers often seek correlations between passive measures and an outcome variable. Therefore, specifying a clear study purpose and a well-defined research question can be useful to limit the number of data patterns to be explored (Höfler et al., 2023). To make the research process transparent, the preregistration should indicate which part of the study is exploratory and which part is confirmatory (Höfler et al., 2023).

A common study purpose among researchers utilizing digital phenotyping data is developing a prediction model to predict a certain outcome. The primary goal is to develop a new prediction model, validate a prediction model, or both (Collins et al., 2015; Velozo et al., 2022). Studies aiming to develop a new prediction model typically involve some form of validation, often through the use of training and testing sets.

##### Study design

The research methodology of a study can be further classified into two primary categories: idiographic and nomothetic (individualized vs. non-individualized). In nomothetic approaches, researchers aim to make general observations/predictions about the population under study. In contrast, idiographic approaches target predictions customized to individuals over time (Molenaar, 2004; Molenaar & Campbell, 2009).

#### Operationalization of main constructs

##### Main constructs of interest

The importance of conceptual clarity is widely acknowledged in psychological research (Bringmann et al., 2022). Conceptual clarity can be difficult to achieve when utilizing passive smartphone measures, where it is not always evident what is measured (Davidson, 2022). Researchers must reflect on the main constructs of interest and define how they are operationalized.

##### Assumptions

When using passive smartphone measures, researchers often make certain assumptions about what each measure represents. For example, previous studies used GPS data as a measure of social behavior. However, it is not yet clear which part of the social behaviors these measures capture and how accurately they capture the intended construct (Langener et al., 2023; Tsapeli & Musolesi, 2015). Often these assumptions are left implicit, making it difficult to build on prior research (Langener et al., 2023). In addition, it is often unclear which data should be used as ground truth, making validation studies challenging (Roos et al., 2023).

To ensure robustness and a proper understanding of study results, it is necessary to know the constructs that passive measures are meant to capture and to validate assumptions about what passive measures capture. While conducting validation studies is a research objective on its own, it can be a good starting point to refer to previous studies that have examined the relationship between the passive measures and the constructs of interest. Using constructs that are operationalized in the same way across studies will help to compare the results of different studies (Huckvale et al., 2019).

### Data collection and sampling plan

Several decisions come into play during data collection, potentially influencing the study outcome. This section delves into aspects such as the selection of devices and sensors, the frequency of data collection, and the methodology used for participant recruitment (see Textbox 2 for all proposed elements, with those discussed in greater detail highlighted in bold).

**Textbox 2** Proposed elements to preregister in the section “Data collection and sampling plan”.

**Table:** 

• Study context
o **Time and location**
o Study duration
• Device and sensor
o **Devices**
o **Sensors**
• **Sampling strategy**
• Participants
o **Recruitment and study procedure**
o Impact of your study
o **Sample composition**
o **Sample size**
o Stopping rule
o **Outreach to the participants**
• Data export, storage, and sharing
o **Data export**
o **Data storage**
o Data sharing (see *Replicability and open science practices*)
• **Other data streams**
• **Secondary data analysis/using existing datasets**
o Time of preregistration
o Explanation of existing data
o Knowledge about existing data
o Prior analysis performed

Bold topics are further detailed in this tutorial paper.

#### Study context

##### Time and location

Different environments and seasonality might have an impact on the collected data. For example, GPS or accelerometer data will probably differ if they were collected in a warm area relative to a cold area, because people may behave differently in warm areas (Mohr et al., 2017). Additionally, the variable of interest can be affected by seasonal effects (Digital Sensing Workshop Participants, Workgroup 3, 2023). To the extent that location data are informed by cell towers, they can also be less precise in rural areas or where there is otherwise poor coverage.

#### Device and sensor

##### Devices

When using passive smartphone measures, researchers usually use different devices to collect the data. This decision is often driven by practical considerations, such as the compatibility of data collection apps only with Android devices, thereby excluding the use of iOS devices. The quality of data may vary across different devices, and suboptimal devices could introduce measurement error (Nelson et al., 2020; Velozo et al., 2022). Moreover, variations between devices can impact the interpretability of the collected data (Davidson, 2022; Nelson et al., 2020).

In some studies, participants use their personal smartphones, while in others, they are provided with a smartphone (Harari et al., 2016). While providing smartphones might enhance data quality and standardize measures across participants, it could impose an additional burden on the participants. Furthermore, participants may use a provided device differently from their own smartphone, potentially influencing the results. Additionally, sometimes participants use multiple phones during the study or switch their phones during the study period, which could introduce biases, as usually data from only one phone of a participant are collected during the study and should be checked by the researcher.

##### Sensors

Researchers often use a variety of sensors to collect data (e.g., GPS, Bluetooth, or Wi-Fi), and multiple sources can even be used to collect the same information. For example, if location data are the focus of the study, the native smartphone GPS might be used, along with additional data exported from the Google Maps timeline. In such cases, the exact source of the data collection may not be known to the researcher. Google, for example, uses nearby Wi-Fi networks, cell towers, and GPS for location services, which adds complexity to understanding data sources.

Measurement errors can arise in data collection depending on the app and sensors used (Davidson, 2022). Researchers should assess whether the chosen sensors and/or app(s) used have been validated (Hicks et al., 2019). In the preregistration, researchers are invited to reflect on the estimated reliability and accuracy of the measures (Davidson, 2022; Nelson et al., 2020), and to justify why this level of reliability is suitable for the study design (Nelson et al., 2020). While there are currently no standardized norms in the field for these evaluations, reflecting on the reliability of measures represents a proactive step toward establishing standards.

#### Sampling strategy

Passive smartphone measures can be continuously captured throughout the day. However, collecting data continuously, such as GPS coordinates, often leads to fast battery drain (Velozo et al., 2022). Consequently, researchers frequently decide to adopt alternative data collection approaches—by sampling at specific time intervals, when particular events occur, or a combination of both (Wrzus & Schoedel, 2023).

When researchers opt for data sampling, one common strategy is to establish a fixed time interval, such as every 10 min, for data collection. Choosing an appropriate time scale requires careful consideration of the expected fluctuations in the measured variables (Velozo et al., 2022). This determination can be made through theoretical reasoning or by conducting a pilot study (Velozo et al., 2022). A widely used guideline suggests that the sampling frequency should be at least twice the frequency of the smallest expected fluctuation in the variable of interest (Bogdan, 2009), especially when considering that sampling frequency may be less than anticipated due to pushback from the underlying operating system (Currey & Torous, 2023; Niemeijer et al., 2023). Nevertheless, when deciding on a sampling frequency, the researcher should consider practical constraints, such as battery drain, which could reduce participant retention.

On top of that, researchers frequently employ event-triggered data collection. This method involves collecting data based on specific events or participant actions. For instance, during periods of increased movement, GPS coordinates may be recorded more frequently than when the participant remains at a particular location for an extended period.

A specific form of event-triggered data collection can be used when combining passive sensing with ESM (see also section *Other data streams*). When ESM and passive sensing are combined, passive sensing can be used to prompt participants to complete their ESM questionnaires. For instance, researchers can utilize passive sensing to trigger questionnaires based on specific contexts or behaviors of interest, such as drinking behavior. GPS data, for example, can be employed to automatically initiate a questionnaire when the participant enters a bar and is thus likely to drink (Ebner-Priemer & Santangelo, 2024).

#### Participants

##### Recruitment and study procedure

Passive smartphone measures present a hurdle for participant recruitment because often sensitive information, such as GPS data, is collected and battery drain may increase. As a result, participating in such studies could be burdensome for some individuals. Therefore, building trust among the population being studied becomes a critical consideration. Some individuals may be hesitant to participate in a study involving such data, leading to potential biases in the studied population (Wrzus & Schoedel, 2023).

##### Sample composition

Furthermore, within a sample, heterogeneity often exists. Factors such as demographics and lifestyles can influence how people interact with their smartphones and how often they carry their phone with them. Given that many studies have limited sample sizes, they may struggle to account for this diversity. To address this, researchers should define their study sample based on their specific research question. When recruiting participants, researchers must evaluate how representative their sample is of the population that they aim to generalize to. It is crucial to assess whether the selected groups in the study adequately represent the diversity and characteristics of the population being studied (Digital Sensing Workshop Participants, Workgroup 3, 2023). One strategy for obtaining a more diverse sample might be to offer the study in different languages and to have a contact person for participants to contact throughout the study.

##### Sample size

In line with this, researchers must address the potential statistical inference issues that may arise when working with a small sample size—characterized by a small number of participants and/or a restricted number of measurement time points. A study with insufficient sample size could lead to underpowered results, diminishing the ability to detect meaningful effects (Davidson, 2022). Therefore, the sample size chosen and why it is appropriate to effectively answer the research question should be justified (Collins et al., 2015). When determining the sample size, it is helpful to consider the number and distribution of variables and the expected measurement error of the sensors used (Digital Sensing Workshop Participants, Workgroup 3, 2023), as well as doing a power analysis (Lafit et al., 2021).

##### Outreach to the participants

Researchers often reach out to participants during the study. This is particularly relevant in intervention studies where researchers may engage with participants to implement specific interventions. However, outreach may also be necessary even in nonintervention studies, such as when dealing with missing data to enhance adherence (Digital Sensing Workshop Participants, Workgroup 3, 2023). Reaching out to participants might (unintentionally) impact the measured outcome (Digital Sensing Workshop Participants, Workgroup 3, 2023) and lead to behavior change. For instance, participants might alter their behavior if they feel observed based on the feedback received during outreach.

Furthermore, sometimes collected data are shared with participants, which might impact their behavior (Davidson, 2022). If a commercial app is utilized for data collection, complete avoidance of data sharing with participants may be impossible, as they can often assess parts of their data through the app itself in a participant-friendly dashboard.

#### Data export, storage, and sharing

##### Data export

Different strategies exist for exporting the collected data. Some studies may require participants to manually send their data to the research team, introducing potential human error or biases. In contrast, in other studies, data export is automated through the data collection app, offering a more standardized approach.

##### Data storage

Passive smartphone measures present a challenge in terms of privacy and security due to the lack of adequate regulations (Davidson, 2022; Velozo et al., 2022). Thus, it should be actively addressed how the data are stored in a safe way (Huckvale et al., 2019; Jagesar et al., 2021; Mulder et al., 2018). Often researchers also decide to anonymize their data, which will be discussed in the section *Data cleaning—anonymization*. Another consideration here is that if data are uploaded to the cloud, the location of the servers could be in one or multiple other countries, which can require data processing agreements to be in place.

#### Other data streams

Researchers often integrate various data types alongside passive smartphone measures. When gathering additional data, it is essential to specify all the types of data collected and the conceptual relationship between the passive smartphone measures and other data sources.

For instance, a researcher might choose to administer pre- and post-questionnaires to assess clinical symptoms or psychological disorders, aiming to examine the differences in passive smartphone measures between distinct groups (e.g., Jongs et al., 2020). Moreover, researchers may use the experience sampling method (ESM) to actively capture symptoms and individuals' perceptions of specific situations throughout the day. When employing ESM, researchers may also benefit from adhering to the *(Pre)registration template for ESM research* (for more information see Kirtley et al., 2021)*.*

#### Secondary data analysis/using existing datasets

When using digital phenotyping, large data sets are often collected and used for multiple studies. When conducting analyses on existing datasets, researchers must be transparent about the data they have previously encountered and whether any analyses have been performed on them (for more information see also van den Akker et al., 2021). Researchers should openly acknowledge their familiarity with the dataset, especially those aspects that are relevant to their hypotheses. There are some scenarios that crosscut the distinction between preregistration before data are created and post-registering secondary data analyses (Kirtley et al., 2021). For instance, when a study project is designed to address not one single but multiple research questions, it may be too impractical to write one extensive registration. Users of the template may opt to preregister methods and primary hypotheses before data collection, and add further co-registrations of analyses planned after data collection has started but relevant trends in the data related to the hypotheses remain unknown (Benning et al., 2019).

### Data processing and feature engineering

Passive sensing collects a large amount of unstructured and private sensitive data, often requiring preprocessing before analysis. In this section, we delve into decisions to be made during feature creation, data anonymization, and quality control (see Textbox 3 for all proposed elements, with those discussed in greater detail highlighted in bold).

**Textbox 3** Proposed elements to preregister in the section “Data processing and feature engineering”.

**Table:** 

• Feature creation
o **Automatically generated summary measures**
o **Summary measures calculated by the researcher**
o **Aggregation choices to compute features**
o Overview of variables
• **Data anonymization**
• **Quality control**

Bold topics are further detailed in this tutorial paper.

#### Feature creation

##### Automatically generated summary measures

Often the easiest data to gain access to and begin analyzing are the daily reported features by the digital sensing device developers. For example, in Apple’s ecosystem, the “Health App” can report measures such as daily activity level, sleep data annotated with sleep stages, resting heart rate, and more. These features are heavily processed from the raw data streams utilizing algorithms that researchers will likely never gain access to. Moreover, the algorithms used to develop these features can change over time, even within the course of a single study, heavily influencing the resulting feature values (Mohr et al., 2017). To enable reproducibility, it is critical to document where a given data stream is derived from, any software or application versions associated with it, and where on the spectrum of minimally processed data to predefined data summaries a given set of features lie (Onnela, 2021; Velozo et al., 2022). These steps are not always possible given proprietary restrictions from companies producing consumer devices, but should be done to the best of a researcher's ability. For future studies, researchers should also aim to assess the validity of exported variables.

##### Summary measures calculated by the researcher

The more common use case, however, is that researchers do not access data that have already been preprocessed on the device (i.e., online), but must do the preprocessing themselves, i.e., offline after the data collection. This step is often called feature extraction and, depending on the types of data to be preprocessed, requires many decisions by the researchers. For example, when working with app usage data, researchers are usually not interested in a single type of app (such as WhatsApp, Instagram, or TikTok), but in broader, psychologically meaningful categories (e.g., communication and social media). Accordingly, researchers have to categorize individual apps in a first step (Sust et al., 2023). It is best to specify in the preregistration whether a ready-made schema (e.g., Schoedel et al., 2022) is to be used or a new, individual scheme is to be created. Another example is the preprocessing of GPS data, which are often stored in raw form as latitude and longitude (e.g., 48.156016, 11.583221). Researchers usually enrich these coordinates in a first step with information from location databases (e.g., HERE, OpenStreetMap, Google Maps) to extract indicators for mobility behaviors, such as places visited (e.g., restaurants, cafés, shops, etc.; see Müller et al., 2022, for a tutorial on GPS data processing). Here again, researchers are confronted with many questions such as in which radius of the raw coordinates to search for places or what to do if several places fall within this radius—if, for example, a store, a hairdresser, and a café can be found at the same address. In addition, depending on the location database used, individual places might again have to be categorized (for empirical illustration, see e.g. Schoedel et al., 2023).

We think that the two examples illustrate that preprocessing can become arbitrarily complex, especially as there may additionally be anomalies in the data that researchers might not foresee during the preregistration phase. Nevertheless, we argue for specifying as many decisions as possible in the preregistration. One helpful approach in this context is to collect pilot data on single persons and to set up the preprocessing pipeline before preregistration. This not only helps to specify some researcher's degree of freedom in advance, but also has the positive side effect of explicitly checking the data quality before the data collection. In this context, researchers can also fall back on standardized feature extraction frameworks that can relieve some of the preprocessing work (e.g., DBDP,^2^ Open mHealth,^3^ Rapids^4^).

##### Aggregation choices to compute features

When computing certain variables, researchers have to decide on aggregation methods, such as a specific time window. For instance, GPS coordinates are transformed into measures that reflect how much time an individual has spent at certain places (e.g., a restaurant) within the past hour or day. This process involves selecting a time window to aggregate passive data, spanning from brief intervals of a few minutes to more extended durations of weeks. This also becomes relevant when combining passive sensing with other data streams (e.g., questionnaires) that are measured on a different time scale (for more information see Velozo et al., 2022). The choice of the time window must align with theoretical and conceptual considerations, which means that researchers must ensure that the selected time scale matches the intended construct that they aim to measure (for more information see Langener et al., 2024a, 2024b). Previous research has shown that the time scale chosen to summarize variables can affect the results of the analysis (Cai et al., 2018; Langener et al., 2024a, 2024b; Schoedel et al., 2020; Sun et al., 2023).

At the same time, researchers face the decision of which method to employ in calculating features within a specified time interval. For example, when determining the time spent at a restaurant during the day, researchers might opt to calculate the sum of durations or compute an average using metrics such as the median or mean, or more robust alternatives like the Huber mean (Huber, 1992).

#### Data anonymization

Data anonymization is important for enabling the sharing of collected data publicly or for compliance with regulatory guidelines like the European Union General Data Protection Regulation (EU-GDPR). However, passive smartphone measures collect a variety of private and sensitive information, presenting challenges for both data storage and anonymization (Onnela, 2021). If possible, identifying information should be removed from the data. For example, Wi-Fi connections should be replaced with an anonymized key (for more information on data storage and anonymization see Jagesar et al., 2021). For GPS data, a random error can be added, the location data can be centered around a centroid, or sensitive locations can be masked. In addition, location data can be labeled—enriched with more information, such as whether the participant was in a bar or a restaurant (this should be done before anonymization).

Some data sources, such as heart rate data, are an indicator of health status, so they are considered special category data (according to the EU-GDPR regulation). Similarly, to the extent that GPS locations can reveal special category data (e.g., church visits indicating religious beliefs), app usage patterns can also reveal personal or special category data (e.g., type of dating app indicating sexual orientation). Overall, free-text data (including app names), audio data, photo data, and GPS data are particularly likely to be identifiable, and each stream should be treated with appropriate care.

Researchers should also consider what level of anonymization is needed for their data. For example, data that will be made available to other researchers (i.e., open access) may require additional steps to ensure anonymization, such as replacing timestamps.

A priori approaches to anonymization may be preregistered, without knowing the exact de-identifying data manipulations in advance. In such cases, the preregistration may refer to the respective decision body (e.g., study advisory board, institutional review board) or process (e.g., regular anonymization reviews) that will determine final measures that apply to data collection and data sharing.

#### Quality control

Digital phenotyping data are often unstandardized and noisy (Huckvale et al., 2019). For example, technical errors might lead to implausible data (Onnela, 2021). Therefore, researchers should indicate which quality control checks they conduct and how to manage the existence of unrealistic data and outliers.

A good initial step is to verify whether the defined sampling scheme is fulfilled. For instance, if a sensor was sampled every minute, it should be verified whether this was actually carried out. If the sensor is sampled more or less frequently, researchers should specify the method of handling it, such as computing the mean value or indicating missingness.

Labeling data as missing is common in digital phenotyping studies because it is not immediately obvious when data are missing. For example, measures such as app usage may be zero because no app was used or because the app used to collect the data crashed. Therefore, researchers often come up with strategies to exclude data (i.e., labeled as missing). Such strategies are, for example, to include a day's data only if at least half of that day's data are recorded (Nickels et al., 2021). The strategy chosen to label missing data has been shown to affect the outcome of analyses and should therefore be preregistered (Langener et al., 2024a, 2024b).

Another step is to check for unrealistic data points, such as excessively high step counts, or GPS artifacts, such as erroneous GPS points in a sequence. It can be difficult or impossible to establish a universal rule for handling outliers, as it can vary from study to study (Nelson et al., 2020). Nevertheless, transparency is crucial in disclosing the procedures and reasons for excluding data. It should be specified whether outliers are eliminated due to technical impossibilities and clearly evident artifacts or for statistical robustness. Some exclusions may occur after the data analysis, which were not included in the preregistration due to unforeseen circumstances. In such cases, this should be reported in the main manuscript.

### Missing data handling (absence of data)

Missing data is a common challenge in smartphone sensing studies, arising from a variety of sources including sensor limitations, participant behavior, and connectivity issues. Sensor limitations, such as the restricted range of detectable input (e.g., the sensor range of Bluetooth proximity detection), often lead to data gaps. Moreover, participant non-compliance, such as insufficient charging or dictating messages rather than typing, culminates in lapses in data collection. Connectivity problems also interfere with both the transmission and storage of data. Patterns of missingness may introduce further complications when data are merged from heterogeneous sources, which may be intricately linked to the research question itself. For instance, comparing rates of non-collection in iOS and Android devices, Kiang et al. (2021) showcase how distinct missingness patterns may reflect selection bias tied to different sociodemographic characteristics. These limitations are always going to co-occur and interfere with data collection when using a smartphone as a means to collect data. As a result of not considering missing data, reliable and valid conclusions can be difficult to draw, as statistical power is reduced, biases to parameter estimates are introduced, and most importantly, we ignore specific behaviors that may provide additional insight into the phenomena of interest (Woods et al., 2023). In turn, the reliability and generalizability of inferences and findings become irreproducible (Button et al., 2013). Thus, how missing data are handled is an important part of the preregistration (see Textbox 4 for all proposed elements, with those discussed in greater detail highlighted in bold).

**Textbox 4** Proposed elements to preregister in the section “Missing data handling (absence of data)”.

**Table:** 

• **Expectation of missingness**
• **Identification of missingness**
• **Handling missing data**

Bold topics are further detailed in this tutorial paper.

#### Expectation of missingness

Hence, users of our template are invited to declare all plausible sources and expected rates of missing data. Such information is ideally drawn from prior piloting or published reports.

#### Identification of missingness

Next, the preregistration should inform about all related a priori decisions, specifically, how and at which thresholds missing data points will be identified (Hicks et al., 2019). This can be focused on a distinction between the missing data mechanisms where it can be completely at random, missing at random, and missing not at random. An example of missing not at random would be incomplete data due to low battery state, which is likely, on average, to occur later in the day.

#### Handling missing data

As a result of these distinctions, it can allow us to specify how missing data will be handled, if a reduced (e.g. complete-case) dataset will be used, or if missing data will be imputed. Given the multitude of different imputation methods and related techniques (e.g., multiple imputation, missing indicator method, modeling weights based on missing data), the development of an appropriate imputation model and diagnostic checks can be guided by the growing body of literature to avoid introducing bias (e.g., Nguyen et al., 2021; Woods et al., 2023). The preregistration can also be used to clarify which amounts of available data are considered necessary for conducting the primary analytical approach and which alternatives are planned if less data is obtained.

### Data analysis

Machine learning models are frequently employed for analyzing the large amount of data collected in digital phenotyping studies. Consequently, the primary emphasis of this section is on the decisions involved in applying machine learning models to digital phenotyping data, with only brief discussions on other analytical topics (see Textbox 5 for all proposed elements, with those discussed in greater detail highlighted in bold). We assume that researchers using this tutorial possess some basic knowledge of machine learning; for those without such knowledge, we recommend introductory readings on the topic (for example, Pargent et al., 2023, for supervised machine learning).

**Textbox 5** Proposed elements to preregister in the section “Data analysis”.

**Table:** 

**•** Machine learning models
o **Task**
o **Cross-validation setup**
o **Performance evaluation**
o **Machine learning algorithm**
o **Feature selection**
**•** Other statistical analysis
o **Statistical model**
o **Multilevel model**
o Analytical problems
o Inference criteria
**•** Transformation of variables
**• Sensitivity and multiverse analysis**
**• Exploratory analysis**

Bold topics are further detailed in this tutorial paper.

An important consideration when choosing a data analysis approach is whether the research goal is idiographic or nomothetic, as this will influence which analytical methods are suitable. In the context of machine learning models, the decision between these approaches influences the selection of CV strategies. For nomothetic modeling, it is advisable for the training and test sets to consist of different participants (Saeb et al., 2016); otherwise the model may generalize less well to a new population. However, depending on the specific application scenario, researchers may choose a CV setup wherein participants overlap between the training and test sets. This approach might be preferred, for instance, when developing an ongoing intervention for a particular population. In such cases, the potential limitation in generalizability should be discussed. For idiographic modeling, it is important to take the temporal order of the data into account (see *Prediction task and cross-validation setup* section).

If researchers are interested in doing other statistical analysis, numerous methods are available for analyzing data from nomothetic and idiographic perspectives, or combining both. As previously mentioned, the ESM preregistration template is a good starting point for delving deeper into statistical analyses, particularly for nomothetic models. For idiographic methods, Piccirillo and Rodebaugh (2019) provide a comprehensive overview, reviewing key statistical methods in psychology, with relevance extending to other fields.

Regardless of which way is chosen to analyze the data, researchers should make sure that the method aligns with their idiographic or nomothetic research focus.

#### Machine learning models

##### Prediction task and cross-validation setup

Machine learning often follows an exploratory approach, rather than testing predefined hypotheses as in classical confirmatory statistics. The aim commonly is to develop models that can make generalizable predictions on new data or discover novel patterns. However, whether confirmatory or exploratory, properly partitioning data for training and evaluation sets allows for an unbiased estimate of model performance. Out-of-sample validation supports robust evaluation of a predictive model’s ability to generalize to unseen data. Overfitting can occur when researchers "peek" at the test data or if information "leaks" between partitions. Preregistration strategies can enhance integrity, even for exploratory machine learning.

Machine learning competitions can support unbiased evaluations of machine learning methods. Platforms like Kaggle provide standardized datasets where competitors submit predictions for held-out test observations that the platform evaluates, comparing performance metrics without exposing the ground truth labels. This procedure ensures that competitors have equal access to train data while guaranteeing that overfitting to the test set cannot occur, providing an unbiased evaluation. Researchers can adopt similar strategies by preregistering how they will partition available data between training (model development) and validation (performance assessment) sets. Preregistering these procedures promotes transparency about researcher degrees of freedom while preventing questionable tactics.

In addition to robust partitioning procedures, strategies that limit post hoc modifications following observation of test set performance should be included in preregistered analysis plans. Using separate individuals/teams to prepare predictions versus evaluate results provides additional insurance against bias. This approach is analogous to clinical trials where researchers evaluating patient outcomes remain blinded to patient treatment assignments to prevent their expectations from inadvertently influencing results. While absolute isolation between teams may prove logistically infeasible for smaller studies, an emphasis on evaluating preregistered machine learning pipelines in an automated fashion provides a degree of methodological safeguarding as well. For example, analysis plans could specify details of cross-validation (CV) schemes, like using a nested tenfold validation approach with 10 inner loops for hyperparameter tuning and 10 outer loops for performance estimation, relying on a predefined random seed of 29,736 for reproducibility. Overall, as with large-scale modeling competitions, preregistration offers clear benefits for exploratory machine learning analyses by minimizing analytical flexibility that could undermine the validity of performance assessments.

One classic approach to out-of-sample validation is the train–test split. While straightforward, this method has the drawback of not using the entire dataset for training, potentially leading to suboptimal model performance, particularly in smaller datasets. Alternatively, CV maximizes data utilization, offering a more reliable performance estimate, especially in the case of limited data.

In the context of time series data, such as sensing data, traditional *k*-fold CV, which involves random splitting into *k* equal parts, is often inadequate due to the sequential nature of the data. Traditional *k*-fold CV—which involves randomly splitting a dataset into *k* equal parts—offers inadequate protection for time series data (e.g., passive sensing) as the random splitting can lead to time leakage in which the model inadvertently learns from future data. *Blocked k*-fold CV can offer a solution, where data are divided into larger, contiguous blocks, maintaining chronological order and breaking temporal dependencies (Bergmeir et al., 2018). Alternatively, a sliding time window or rolling origin approach (Bergmeir & Benítez, 2012) can be used, dividing the data based on a time component. Selecting the appropriate window size is critical and should be guided by the specific characteristics of the data and the underlying temporal patterns.

Another challenge in CV is the risk of data leakage during feature extraction. For instance, if feature scaling (i.e., normalizing the variable) is performed using the range of the entire dataset, information from the test set could accidentally influence the training process. To prevent this, feature processing should be conducted independently within each fold of the CV loop.

Similarly, adjusting the hyperparameters of a model is typically achieved through out-of-sample validation. For example, in random forest models we wish to tune hyperparameters that describe the number of trees and the depth of each tree (Breiman, 2001). These hyperparameters are tailored to the training dataset, necessitating their calibration within each cycle of the CV loop. This process forms a nested loop, which is computationally demanding. Therefore, it requires significant planning and consideration for resource allocation, especially in large-scale studies or when working with complex models.

##### Performance evaluation

Effective and transparent performance evaluation is at the core of predictive modeling. Central to this process is selecting appropriate evaluation metrics and reporting on them consistently according to the TRIPOD guidelines (Collins et al., 2015). For regression models, *R*^2^ and mean squared error (MSE; or variations thereof) are commonly used, although it is currently unclear how *R*^2^ should be calculated in a multilevel setting (Piepho, 2023; Rights & Sterba, 2023). In classification tasks, common metrics include accuracy, sensitivity, and specificity. However, in cases of severe class imbalance, more robust measures like the area under the receiver operating characteristic (ROC) curve or the Matthews correlation coefficient are recommended (Halimu et al., 2019).

Furthermore, researchers should not only register the measures for assessing performance but also predefine their interpretation of plausible results. This means establishing clear criteria for what constitutes “good” performance and setting acceptable levels of uncertainty. For example, in the context of depression detection, it is important to determine how many false positives are tolerable for use in clinical practice (Mohr et al., 2017). One way of doing this is by creating benchmark models that the predictive models can be compared to. These could be as simple as an intercept-only model (representing an average) or models using “free” information that is readily available, like those based on temporal factors (e.g., time of day). Such relative measures can enhance the transparency in evaluating complex predictive models against those using very little information.

##### Machine learning algorithm

The decision-making involved in algorithm selection and model specification for digital phenotyping data becomes even more complex in the context of machine learning and other advanced modeling approaches. Although initial guidance can be found in reporting protocols such as TRIPOD (Collins et al., 2015), the development of TRIPOD-AI (for artificial intelligence; Collins et al., 2024), TRIPOD-Cluster (for clustered data; Debray et al., 2023), and TRIPOD-P (for healthcare applications; Dhiman et al., 2023) demonstrates that existing frameworks often fail to address challenges that arise in new areas such as digital phenotyping.

One key decision facing researchers modeling digital phenotyping data is whether to use a supervised or an unsupervised modeling approach. Often the research question itself provides insights into whether a supervised or unsupervised machine learning algorithm will be employed. Supervised learning approaches are designed to predict a target outcome, given a set of inputs. Examples of these approaches include elastic net regularized regression, random forests, and Bayesian additive regression trees. Unsupervised learning approaches do not specify an outcome and instead identify patterns within a given set of inputs (Hastie et al., 2009). In digital phenotyping studies, a combination of both approaches may be employed due to the substantial amount of data collected. Thus, unsupervised machine learning models may be utilized to reduce the number of predictors (see the section *Feature selection* below).

Another type of machine learning is reinforcement learning, in which an “agent” learns the optimal behavior in an environment to receive the maximum reward (Kaelbling et al., 1996). When applying reinforcement learning to digital phenotyping data, researchers should specify the use of active versus passive reinforcement learning methods and provide justification for their selection. In active learning, an agent makes choices in order to optimize outcomes based on its changing environment. In passive learning, the agent follows and evaluates the performance of a pre-programmed algorithm or fixed set of rules, without making choices. Passive learning may be suitable with larger amounts of labeled data, whereas active learning can be an effective alternative when there is less available data or if this is difficult to acquire.

##### Feature selection

Several approaches exist for selecting features to include in a machine learning model. Feature selection is often partially theory-driven, as when researchers include features that make sense from a theoretical point of view. In addition, researchers often use data-driven approaches for selecting which features are retained in the final model. To avoid violating the independence of the training and test, researchers employing data-driven approaches must use only the training data to inform feature selection.

Filtering techniques are often used in feature selection, as when redundant features are removed based on their high correlation in the training data. Another common step is to remove features with zero or near-zero variance. Such feature selection techniques are, for example, implemented in the *caret* package in R and can be easily applied by researchers (Kuhn, 2008).

Some machine learning models have built-in feature selection techniques. For example, LASSO [least absolute shrinkage and selection operator] regression can handle unimportant features by shrinking the beta coefficient to zero (Tibshirani, 1996). In addition, decision trees assign importance to features based on how often they are used for partitioning. Decision trees can also be pruned to avoid overfitting, implicitly selecting the most important nodes/features and removing unimportant features (Pargent et al., 2023).

Another technique is feature/dimensionality reduction. Dimensionality reduction approaches represent a class of unsupervised learning methods that have increased relevance for digital phenotyping studies (Barnett et al., 2018). Intensive longitudinal data can result in a large number of candidate variables (derived features), such as when aggregating (e.g., mean, *SD*, range, kurtosis), or when using model parameters from networks. Additionally, there is often correlation among possible predictors, due to multiple engineered features relying on the same raw data sensor stream, and because many features are generated for associated constructs (e.g., active minutes, calories burned, heart rate). Lastly, the relative infancy of our collective knowledge about digital phenotyping means that researchers often lack guidance about which sensors or derived features to include. Dimensionality reduction approaches can support the identification of new patterns among model inputs and are thus well suited to advancing current knowledge. For example, principal component analysis can be used to reduce the number of features included in the final model. The reduced number of features will be uncorrelated with each other, but will still retain the essential information from the original set of features (Wold et al., 1987).

#### Other statistical analysis

##### Statistical model

In addition to machine learning pipelines, researchers using passive sensing are sometimes interested in using other statistical analyses to describe and model relationships in the data. These may range from simple bivariate correlations to more complex techniques such as multilevel modeling or network analysis (Barnett et al., 2018; daSilva et al., 2021; Lekkas et al., 2022).

##### Multilevel model

The ESM preregistration template (Kirtley et al., 2021) contains several useful questions to help specify multilevel models for time series data. The conclusions that can be drawn from such a model depend critically on several decisions made by researchers, such as how input features are created (see section *Data processing and feature engineering*), what outcomes are of interest, and how variables and models are selected in the final analysis. In particular, researchers should be aware of the additional complexities that may arise due to the temporal structure of the data, such as irregularly spaced measurements, unequal sampling frequencies of different variables, and the potential autocorrelation in the data. Further, it has been recently argued that adopting a more flexible complexity approach may better model the dynamic temporal relationships that cannot be examined in generalized linear mixed models (Hasselman & Bosman, 2020).

#### Sensitivity analysis and multiverse analysis

As emphasized repeatedly, researchers face many critical decisions during a passive sensing study for which no clear guidelines exist (Langener et al., 2024a, 2024b). For example, there may be many reasonable ways to aggregate data or to handle missing values. While most studies focus on reporting the results of a single analysis pipeline, a multitude of equally plausible alternatives allow for significant researcher degrees of freedom. This “multiverse” of choices (Steegen et al., 2016) involves exploring every possible option, thereby enabling an analysis of the effects of differing choices, which improves the robustness of the research results. Researchers should thus outline steps to assess how analytical decisions influence their findings.

#### Exploratory analysis

If there are any intentions to perform additional exploratory analyses beyond the scope of what has explicitly been preregistered, it is essential to elucidate the objectives and rationale behind these additional analyses. Clarity in defining the goals of these exploratory efforts is crucial for maintaining transparency and rigor in the study.

Researchers should also explicitly commit that any analyses not detailed in the preregistration will be presented as “exploratory” in the final paper.

### Replicability and open science practices

Open science practices (e.g. data sharing) focus on making data more openly available. Data sharing with other researchers can benefit the field, especially considering the typically small sample sizes and short study durations in current research. Pooling data across studies allows for more robust conclusions and broader generalizability of findings (Huckvale et al., 2019). To promote transparency and collaboration, researchers should reflect on whether they plan to share their data with other researchers and outline the methods they will use to do so in a secure manner (see section *Data cleaning—anonymization*). Researchers should consider what data are shared with whom and the conditions under which the data are reused or redistributed, and should follow the guidelines developed by government and commercial organizations.

As the number of options available for any given analytical approach continues to increase (e.g., Python vs. R, which version of R, which package in R, which version of the package, which settings were selected), so too does the importance of comprehensively reporting these relevant details. In addition to this transparency, threats to reproducibility caused by the complexity associated with data analysis and machine learning can be mitigated through the inclusion of source code. We encourage researchers to openly share their code used for preprocessing, feature engineering, and statistical modeling to enable scientific and computational reproducibility and transparency, as methods sections of publications rarely provide sufficient information to allow for full computational reproducibility. However, there may be limits to sharing code, such as not being allowed to share certain sensitive parts of the code (e.g., code that produces anonymous GPS data may use identifying data).

Further steps to increase reproducibility include more comprehensive solutions such as virtualization, in which platforms like Docker (Merkel, 2014) can be used to containerize the entire analytical environment (analysis program, libraries, dependencies, etc.) into a container package that can then be used by other researchers (Onnela, 2021).

## Discussion

Passive smartphone measures are a powerful tool and are increasingly used in psychological research. However, the use of passive smartphone measures involves a variety of decisions that can lead to different conclusions (Cai et al., 2018; Langener et al., 2024a, 2024b; Niemeijer et al., 2022; Sun et al., 2023) and are not always reported transparently. To help researchers make these decisions and to improve the replicability and reproducibility of passive sensing studies, we developed a preregistration template. We discussed six core themes that are particularly important when working with passive smartphone measures: (1) conceptualization of constructs and the research question, (2) data collection and sampling plan, (3) data processing and feature engineering, (4) missing data handling, (5) data analyses, and (6) replicability as well as open science practices. We believe that by clarifying researchers' degrees of freedom and promoting transparency, the current standards of studies using passive smartphone measures will increase.

### Navigating the garden of forking paths

Within the six core themes we have discussed throughout this paper, there are many choices to be made when using passive smartphone measures, and often researchers are not aware of all of them. Broadly, decisions to be made can be distinguished between “*known knowns*,” which represent what we are aware of and understand; “*known unknowns*,” which represent aspects that are recognized as gaps in our knowledge; and “*unknown unknowns*,” which is information beyond our awareness. This conceptualization is equally applicable to research in a broader context (Logan, 2009). In digital phenotyping studies, *known knowns* are easily identifiable elements such as self-developed algorithms during data processing and feature engineering. *Known unknowns* arise, for example, when researchers use commercial algorithms without access or in collaborative efforts where extensive documentation is impractical. *Unknown unknowns* arise in digital phenotyping studies, for example, when preprocessing steps have been taken that the researcher is not aware of, or when the underlying commercial algorithm changes. These decisions lend themselves in varying degrees to preregistration. *Known knowns* can easily be preregistered by researchers. *Known unknowns* should be explicitly disclosed in a preregistration, being as specific as possible. *Unknown unknowns*, however, are challenging to deal with because we cannot preregister things we do not know. Therefore, future research should aim to establish standards that minimize the *unknown unknowns* to make reporting more transparent.

In addition to the challenge of identifying all possible decisions that need to be made, researchers also face the difficulty of choosing one option over another. Ideally, researchers should not only preregister the choices they make but also justify and explain their decisions. This should be documented in both the preregistration and the paper, allowing other researchers to learn from previous work when faced with similar decisions. We acknowledge that this might not be an easy task, especially as digital phenotyping is a rather new field where substantial debate and consensus about particular decisions does not yet exist. Nevertheless, being open about this uncertainty can be beneficial, helping other researchers understand the decision-making process and potentially identifying new research areas if many researchers struggle with similar decisions and uncertainties.

Given the many choices and uncertainty that researchers face, robustness checks and multiverse analyses are often part of a preregistration offering a systematic approach to exploring various plausible decisions (Steegen et al., 2016). Practical constraints, however, limit the feasibility of testing all potential decisions due to computational expense. To address this, we recommend that researchers report which decisions (e.g., hyperparameters, level of aggregation) they aim to test, and explicitly state whether other decisions would have been equally reasonable even if they cannot be tested. Ideally, researchers should think about which decisions are most important for their research question and try to address those.

This paper delves into the myriad decisions researchers face when working with passive smartphone measures, providing guidance for informed decision-making. To orient the reader through the garden of forking paths of smartphone sensing methods (cf. metaphor by Gelman & Loken, 2013), we draw the attention to the crucial forks that we all encounter rather than imposing a single dogmatic route. However, it is important to acknowledge that some decisions are quite complex. Despite our discussion, the challenge of resolving these complex decisions may remain. To provide further guidance in making informed decisions, Table 1 summarizes some key references for additional resources.

**Table 1:** Key references

Topic	Reference
Using passive measures combined with ESM	*• ESM preregistration template:* Kirtley, O. J., Lafit, G., Achterhof, R., Hiekkaranta, A. P., & Myin-Germeys, I. (2021). Making the black box transparent: A template and tutorial for registration of studies using experience-sampling methods. Advances in Methods and Practices in Psychological Science, 4(1), 2,515,245,920,924,686 *• How to combine both measures:* Velozo, J. D. C., Habets, J., George, S. V., Niemeijer, K., Minaeva, O., Hagemann, N., … & Delespaul, P. (2022). Designing daily-life research combining experience sampling method with parallel data. Psychological Medicine, 1–10
Choosing a time scale to summarize and analyze the data	*•* Langener, A. M., Stulp, G., Jacobson, N. C., Costanzo A., Jagesar, R., Kas, M. J., Bringmann L. F. (2024). It’s all about timing: Exploring different temporal resolutions for analyzing digital phenotyping data. *Advances in Methods and Practices in Psychological Science,* 10.1177/25152459231202677
Applying machine learning models	*• Reporting Guidelines:* Collins et al., (2024). Development of a reporting guideline for diagnostic and prognostic prediction studies based on artificial intelligence (TRIPOD-AI). 10.17605/OSF.IO/ZYACB *• Introduction to Supervised Machine Learning:* Pargent, F., Schoedel, R., & Stachl, C. (2023). Best practices in supervised machine learning: A tutorial for psychologists. Advances in Methods and Practices in Psychological Science, 6(3), 25,152,459,231,162,559
Transparency and reproducibility	*•* Wrzus, C., & Schoedel, R. (2023). Transparency and reproducibility in mobile sensing research. Mobile Sensing in Psychology: Methods and Applications. Guilford Publications *•* Benning, S. D., Bachrach, R. L., Smith, E. A., Freeman, A. J., & Wright, A. G. C. (2019). The registration continuum in clinical science: A guide toward transparent practices. *Journal of Abnormal Psychology*, *128*(6), 528–540. 10.1037/abn0000451
General overview covering multiple topics (book)	*•* Mehl, M. R., Eid, M., Wrzus, C., Harari, G. M., & Ebner-Priemer, U. (Eds.). (2024). Mobile sensing in psychology: Methods and applications. The Guilford Press

### Quality of preregistrations for studies using digital phenotyping

We explored the complexity of decision-making in digital phenotyping studies, emphasizing that researchers may encounter numerous decisions, some of which they may not be fully aware of. Consequently, it becomes crucial to consider the quality requirements when preregistering such studies. Ideally, a good preregistration should be specific, precise, and exhaustive, which means that all steps to be taken are included, those steps are unambiguous, and there is no room left for other steps to be taken (Wicherts et al., 2016).

However, even with our proposed preregistration template, writing such a comprehensive and unambiguous preregistration might remain challenging. Wrzus and Schoedel (2023) suggested that different parts of a preregistration may vary in specificity. For example, for preprocessing choices, the least specific approach would involve the absence of any reporting, a moderately specific level would involve providing an overview of preprocessing decisions, while the highest specificity would involve the integration of alternative preprocessing decisions into the statistical analysis. For confirmatory studies, the ideal is maximum specificity, which requires researchers to be as detailed as possible. For exploratory studies, a lower level of specificity may be more appropriate, provided that it is transparent which decisions were exploratory and which were not.

Overall, we believe that the responsibility for precision in preregistering digital phenotyping studies currently lies with the researcher, requiring them to determine the most suitable level of specificity. As noted by Wrzus and Schoedel (2023), an overly critical attitude toward preregistering digital phenotyping studies could hinder progress, as could overenthusiasm. Nevertheless, in the long run, more standards for preregistering and conducting digital phenotyping studies should be developed and adopted by researchers.

### How to move forward

We have outlined many decisions researchers make while using passive smartphone measures and argued that preregistering them might increase reproducibility. However, improving the reproducibility of research also requires standardizing measures across studies and enabling meaningful comparisons over time. Achieving this goal is challenging, as sensors and machine learning models continue to evolve. As a result, there is a trade-off between using standardized measures and adopting new and potentially better versions. This trade-off is particularly relevant when using passive data in health and clinical applications.

In the context of healthcare and clinical applications, approval by regulatory bodies such as the U.S. Food and Drug Administration (FDA) is typically requisite for market entry. To gain FDA approval, sensors must be verified and validated, meaning that physical parameters must be accurate and precise over time (i.e., verification) and that the outcome, such as a clinical event, is appropriately addressed in the population being studied (i.e., validation, Food & Drug Administration, 2023a). Importantly, if different software versions are used, each should be validated against an older version. Likewise, an objective for future research should be the prevalidation of newly developed algorithms or sensors before their deployment (Food & Drug Administration, 2023c). In the future, we should aim to include such prevalidation as part of the preregistration process.

The FDA has recently introduced a “Predetermined Change Control Plan,” which provides a framework to reflect on potential modifications and changes (2023b). Such a plan includes how changes will be developed, implemented, and validated. It also includes an assessment of the potential benefits and risks. A goal for future research could be to include more consideration of potential—whether intended or unintended—modifications to the sensors or algorithms used, and how these might affect the generalizability for future research results. If standards for passive sensing studies increase, consideration of potential modifications could also be part of the preregistration process.

Our preregistration template provides a resource for reflecting on important decisions, recognizing that these decisions may evolve over time. Given the evolving nature of the field of digital phenotyping, we anticipate that certain decisions will become more or less relevant in the future. Therefore, we consider our template to be a starting point rather than an exhaustive list. Our goal is to update the preregistration template over time, and we invite other researchers to contribute (for more information on how to contribute, see https://osf.io/8k3tm). Overall, we hope that this preregistration is a first step in raising the standards for passive sensing studies.

## Footnotes

1 It is our goal that the template will be included as part of the standard OSF preregistration options in the future.

2 https://dbdp.org

3 https://www.openmhealth.org/

4 https://www.rapids.science/1.9/

## Glossary

Digital phenotyping: Using data from personal digital devices to build a detailed picture of an individual's behavior and health. Simply put, a digital footprint that allows researchers and clinicians to understand patterns, tailor interventions to the individual, and predict future changes. In this paper, we use digital phenotyping interchangeably with *passive sensing*
Experience sampling method (ESM): Consists of structured self-report diary techniques that are used to evaluate mood, symptoms, contextual factors, and personal appraisals as they naturally occur in the real-world environment of participants across time (Myin-Germeys & Kuppens, 2022)
Exploratory vs. confirmatory research: In exploratory research the objective is to find new patterns in the data, subsequently leading to the generation or modification of hypotheses, models, and theories. In contrast, confirmatory research involves the testing of specific hypotheses often using inferential statistics (Höfler et al., 2022)
Feature (creation/engineering): The term *feature* comes from machine learning jargon and is synonymous with the terms *variable* or *predictor*. Feature creation describes the process of how variables are obtained from raw data in data preprocessing. Feature engineering is a more general term that includes feature creation
Idiographic approach vs. nomothetic approach: In nomothetic approaches, researchers aim to make general observations/predictions about the population under study, also often called non-individualized research/models. In contrast, idiographic approaches target predictions customized to individuals over time, also often called individualized research/models (Molenaar, 2004; Molenaar & Campbell, 2009)
Passive (smartphone) measures: Measures collected from smartphones (or other wearable devices). Here we use passive (smartphone) measures interchangeably with *passive data*. Data can be acquired through *sensors* or *phone logs*. Sensors capture various data types. For instance, GPS sensors are used to determine the location, and accelerometers measure movement. Phone log data are derived from interactions with the device but not obtained through a physical sensor (e.g., app usage, text messages, and screen usage)
Preregistration: The researcher publishes the study plan in a time-stamped database such as the Open Science Framework as an immutable document that details the research question/hypotheses, design, and data analysis prior to analyses being conducted (Nosek et al., 2018; van den Akker et al., 2023a, 2023b)
Reproducibility: If a study is reproducible, it means that “consistent results are obtained using the same input data; computational steps, methods, and code; and conditions of analysis” (National Academies of Sciences et al., 2019)
Replicability: Refers to “obtaining consistent results across studies aimed at answering the same scientific question, each of which has obtained its own data” (National Academies of Sciences et al., 2019)
